# The Impact of COVID-19 on Racial and Ethnic Disparities in HIV Pre-Exposure Prophylaxis Retention: Data from a Large U.S. Health Care Organization

**DOI:** 10.3390/ijerph22111677

**Published:** 2025-11-05

**Authors:** Adam C. Sukhija-Cohen, Michael F. Blasingame, Henna Patani, Marie C. D. Stoner, Antón Castellanos-Usigli, Allysha C. Maragh-Bass

**Affiliations:** 1 Center for Health Systems Research, Sutter Health, 795 El Camino Real, Ames Building, Palo Alto, CA 94301, USA; 2 Healthvana, 7162 Beverly Boulevard, Suite 238, Los Angeles, CA 90036, USA; 3 AIDS Healthcare Foundation, 6255 West Sunset Boulevard, 21st Floor, Los Angeles, CA 90028, USA; 4 RTI International, 3040 Cornwallis Road, Research Triangle Park, NC 27709, USA; 5 Duke Global Health Institute, Duke University, 310 Trent Drive, Box 90519, Durham, NC 27710, USA; 6 Center for AIDS Prevention Studies, University of California San Francisco, 550 16th Street, 3rd Floor, San Francisco, CA 94158, USA; 7 Center for AIDS Research, University of North Carolina at Chapel Hill, Lineberger Building, 450 West Drive, Chapel Hill, NC 27599, USA

**Keywords:** HIV, pre-exposure prophylaxis, retention in care, racial disparities, ethnic disparities

## Abstract

Retention in pre-exposure prophylaxis (PrEP) care—defined as receiving a fourth PrEP prescription within 12 months of initiation—remains a major challenge for young adults and individuals minoritized by race and ethnicity in the United States (U.S.), particularly after disruptions in care from the Coronavirus Disease 2019 (COVID-19) pandemic. This study examined changes in PrEP retention before and after COVID-19 among clients ages 18–29 years at AIDS Healthcare Foundation (AHF) Wellness Center clinics across the U.S. We conducted a retrospective analysis of electronic health record (EHR) data from 6047 clients who initiated PrEP between 1 January 2018 and 15 March 2023. Retention was defined as receiving a fourth PrEP prescription within 12 months of initiation. Overall, PrEP initiation increased threefold post-COVID-19, but retention by the fourth prescription declined from 86.2% pre-COVID-19 to 62.6% post-COVID-19 (*p* < 0.001). Clients initiating PrEP post-COVID-19 had significantly lower odds of retention (odds ratio [OR] = 0.13; *p* < 0.001), suggesting these systemic disruptions reduced continuity of care. Additionally, clients who identify as non-White had lower retention odds compared to clients who identify as White post-COVID-19 (OR = 0.80; *p* = 0.003), indicating that racial/ethnic disparities in PrEP care persist beyond the pandemic’s impact. These findings highlight the need for targeted interventions to strengthen retention in PrEP care post-COVID-19.

## 1. Introduction

HIV pre-exposure prophylaxis (PrEP) is the use of highly effective antiretroviral medication to prevent HIV acquisition [1]. According to the Centers for Disease Control and Prevention (CDC), approximately 1.2 million people in the United States (U.S.) could benefit from PrEP. However, as of 2022, just over one-third had been prescribed this HIV prevention medication [2]. While new injectable formulations are now available, the original and most widely available form remains a once-daily oral medication. Clients prescribed daily oral PrEP typically receive a 90-day supply, and CDC guidelines recommend primary care providers offer support and ongoing assessments at least every three months before clients receive their next prescription [1].

Retention in PrEP care after initiation is a public health challenge, particularly among young adults and individuals minoritized by race and ethnicity [3,4,5,6]. Globally, a study of 20 countries/regions found 41% discontinued PrEP within six months of initiation, and among those who continued PrEP, 38% reported suboptimal adherence [7]. It is critical to stay engaged in PrEP care because consistent use is required to sustain the daily oral medication’s protective effect against HIV. Consequently, interruptions in care increase the risk of seroconversion and undermine PrEP’s effectiveness.

The Coronavirus Disease 2019 (COVID-19) pandemic disrupted health care systems in the U.S. and posed major challenges to PrEP services [8,9]. While total PrEP prescriptions have increased annually since before the start of the COVID-19 pandemic, the CDC reports “severe and widening inequities” due to social determinants of health [2], for example, differences by education level and economic stability [10]. Nationally, 94% of people who identify as White who could benefit from PrEP received a prescription in 2022; conversely, only 13% of people who identify as Black and 24% of people who identify as Hispanic or Latino who could benefit from PrEP received a prescription in that same year.

While PrEP uptake among young adults and members of racial/ethnic minority groups has been reported pre- versus post-COVID-19, less is known about the effects of the pandemic on retention in PrEP care. AIDS Healthcare Foundation (AHF) is a global nonprofit organization that provides sexual health services, including PrEP care, across the U.S. This study examines trends in PrEP retention before and after the onset of the COVID-19 pandemic at AHF’s Wellness Center clinics, with a focus on racial/ethnic disparities among young adult clients ages 18–29 years.

## 2. Materials and Methods

Data from clients who (a) were 18–29 years old; and (b) initiated PrEP at Wellness Center clinics operated by AHF were extracted from electronic health records. Unique clients were dichotomized into pre-COVID-19 (from 1 January 2018 to 29 February 2020) and post-COVID-19 (from 1 March 2020 to 15 March 2023). Client data included: (1) PrEP initiation; (2) follow-up PrEP prescriptions (up to the fourth prescription to document one year of daily oral PrEP retention); and (3) self-identified race/ethnicity. PrEP prescriptions were confirmed if they were filled. PrEP retention was defined as receiving a fourth PrEP prescription within 12 months of initiation (yes/no), similar to other studies’ measure of retention [11,12,13]. Clients’ self-identified race/ethnicity were dichotomized into White and non-White, where non-White includes the following categories: American Indian or Alaska Native, Asian, Black or African American, Hispanic or Latino, Native Hawaiian or Other Pacific Islander, or Other. Clients who declined to identify their race/ethnicity are reported but not included in the analyses. Pre-COVID-19 data included 16 AHF Wellness Center clinics in California, Florida, Georgia, Maryland, Ohio, Pennsylvania, and Texas; post-COVID-19 data included 30 AHF Wellness Center clinics in these states, plus Mississippi, Nevada, New York, and Washington, District of Columbia (D.C.).

We fit a logistic regression model to examine whether the odds of receiving a fourth prescription differed by race/ethnicity and whether this association varied between the pre- and post-COVID-19 timeframes. All analyses were conducted using R version 4.4.2.

## 3. Results

Between 1 January 2018 and 15 March 2023, 6047 total clients ages 18–29 years initiated daily oral PrEP at the 30 AHF Wellness Center clinics across 10 states and Washington, D.C. (Table 1). Nearly one-fourth (23.0%) of the total clients each self-identify as White (1390/6047), and 24.7% decline to identify their race/ethnicity (1496/6047). Just over half (52.3%) self-identify as non-White (3161/6047), 

Of the 6047 total clients who initiated PrEP, 1431 (23.7%) initiated pre-COVID-19 (1 January 2018 to 29 February 2020), and 4616 (76.3%) initiated post-COVID-19 (1 March 2020 to 15 March 2023; Table 1). A Pearson chi-squared (χ^2^) test of independence showed that the racial/ethnic distribution of PrEP initiators differed significantly between the pre- and post-COVID-19 periods, with the proportion of non-White initiators increasing more compared to White initiators (χ^2^[1] = 35.80, *p* < 0.001).

Overall, 89.0% of the 6047 total clients who initiated PrEP received a second prescription (5379/6047), 79.4% received a third prescription (4802/6047), and 68.1% received a fourth prescription (4121/6047; Table 2). Pre-COVID-19, 94.9% of the 1431 clients who initiated PrEP received a second prescription (1358/1431), 91.6% received a third (1311/1431), and 86.2% received a fourth (1233/1431). Post-COVID-19, 87.1% of the 4616 clients who initiated PrEP received a second prescription (4021/4616), 75.6% received a third (3491/4616), and 62.6% received a fourth (2888/4616). Retention by the fourth prescription was significantly lower post- versus pre-COVID-19 based on a two-sample proportion z-test (difference = −0.236, 95% confidence interval [CI]: −0.259, −0.213, z = 16.74, *p* < 0.001). This corresponds to 13.8% clients not retained by the fourth prescription pre-COVID-19, compared to 37.4% not retained by the fourth prescription post-COVID-19. 

By race/ethnicity, clients who identify as White had higher overall PrEP retention by the fourth prescription (72.9%; 1013/1390) than clients who identify as non-White (65.9%; 2084/3161; Table 2). These differences by race/ethnicity were present pre- and post-COVID-19. Irrespective of race/ethnicity, all groups’ retention by the fourth prescription declined: 95.5% to 64.4% among clients who identify as White, respectively, and 92.8% to 61.2% among clients who identify as non-White, respectively.

We used logistic regression to examine changes in PrEP retention by the fourth prescription from pre- to post-COVID-19, as well as differences in retention by race/ethnicity (Table 3). The odds of receiving a fourth prescription were markedly lower post- versus pre-COVID-19 (odds ratio [OR] = 0.13, 95% CI: 0.09–0.18, *p* < 0.001). Clients who identify as non-White had 20% lower odds of receiving a fourth prescription than clients who identify as White (OR = 0.80, 95% CI: 0.69–0.93, *p* = 0.003). The race/ethnicity × time period interaction was not statistically significant (OR = 1.34, 95% CI: 0.70–2.58, *p* = 0.379), indicating that the decline in the odds of receiving a fourth prescription pre-COVID-19 versus post-COVID-19 was similar for clients who identify as White and clients who identify as non-White.

## 4. Discussion

The overall purpose of this research was to explore the impact of the global COVID-19 pandemic on the PrEP care continuum among a large cohort of AHF clients ages 18–29 years. We document substantial declines in PrEP retention such that while initiation of PrEP increased threefold post-COVID-19, only 62.6% of clients who initiated PrEP post-COVID-19 received their fourth prescription. This is a sharp reduction compared to 86.2% pre-COVID-19, and attrition continued to affect clients who identify as non-White more than their White peers. Our logistic regression model revealed a significant drop in retention odds post-COVID-19, suggesting the vulnerability of PrEP programs to external disruptions and the need for resilient sexual health care services.

In addition, the lower odds of retention post-COVID-19 among clients who identify as non-White suggests that racial/ethnic disparities in PrEP care are not solely attributable to the COVID-19 pandemic. Structural inequities, such as insurance coverage and stigma, may contribute to these persistent gaps. Lastly, while retention declined overall, the rate of decline did not differ significantly by race/ethnicity. Both clients who identify as White and clients who identify as non-White experienced pandemic-related disruptions; however, clients who identify as non-White started from a lower baseline and remained disproportionately affected.

Studies conducted in the U.S. and globally, during and immediately after the COVID-19 pandemic reported substantial disruptions in PrEP care, contributing to lower retention and widening racial/ethnic disparities in PrEP use [14,15,16]. These findings are consistent with our results, although most of those studies included data only through 2022. Our analysis extends the reach and gravity of this evidence, by showing that such disparities persisted beyond the early pandemic period and remained evident in the first months of 2023.

There are important limitations to acknowledge. First, dichotomizing race/ethnicity into White versus non-White likely obscures critical differences among racial/ethnic subgroups. Future studies should explore retention patterns within more granular categories, something we were not able to explore due to small subgroup sample sizes. Second, we did not stratify by age group; there are developmental differences between 18–24-year-olds and 25–29-year-olds. Third, important factors such as insurance coverage, sexual orientation, geographic location (state or clinic), and pandemic-related service disruptions were not included in the model but may influence retention in PrEP care.

Fourth, data were drawn from a single health care organization, which does not reflect retention patterns in other provider settings or jurisdictions. AHF operates its own pharmacies, which may be unique compared to other health care organizations. In addition, the PrEP care model at AHF during the study period has since evolved in response to new social, political, and medical contexts surrounding PrEP, Therefore, the findings may be less generalizable to the current landscape. Similarly, new PrEP medications became available during the study timeframe, including a new daily oral PrEP formulation in 2019 [17] and the first long-acting injectable PrEP in 2021 [18], which may have increased public awareness and improvements in access to PrEP.

Lastly, using the fourth prescription as a retention marker may not capture AHF clients who discontinued PrEP intentionally or continued PrEP at non-AHF locations. Future studies should consider longer timeframes and multiple institutions to better assess clients’ continuous and long-term engagement in care, along with more nuanced treatment of race/ethnicity data, inclusion of psychosocial factors such as behavioral healthcare needs, and experiences of clients minoritized by other factors such as sexual orientation, gender identity, and/or disability.

## 5. Conclusions

The disparities in PrEP retention by race/ethnicity pre- and post-COVID-19 underscore the need for targeted interventions to improve PrEP care in underserved populations, especially during external disruptions in health care services. The gaps have widened post-COVID-19, and strategies should include telemedicine for PrEP, expanded access to mail-order PrEP medication delivery, culturally tailored and inclusive PrEP care, expanded screening and regular offering of PrEP (without clients have to request it) and policies that reduce cost and logistical burdens for clients facing health care inequities.

That AHF clients who identify as White consistently showed better retention—both pre- and post-COVID-19—confirms the CDC’s findings that universal PrEP access does not ensure equitable outcomes. Efforts to close racial/ethnic gaps in retention are more imperative than ever to achieve the primary national goals set by the Ending the HIV Epidemic in the U.S. These goals include reducing new HIV infections by 90% by 2030 [19]. Addressing these inequities is essential not only for improving PrEP outcomes but also for strengthening the resilience of HIV prevention systems against future global public health crises.

## Figures and Tables

**Table 1:** Number of clients ages 18–29 years who initiated PrEP at AHF by race/ethnicity, pre-COVID-19 versus post-COVID-19.

Demographics	Pre-COVID-19 N (Column %)	Post-COVID-19 N (Column %)	Total N (Column %)
Race/Ethnicity			
White	310 (21.7)	1080 (23.4)	1390 (23.0)
Non-White	475 (33.2)	2686 (58.2)	3161 (52.3)
Unknown Race/Ethnicity	646 (45.1)	850 (18.4)	1496 (24.7)
Total (Row %)	1431 (23.7)	4616 (76.3)	6047 (100.0)

**Table 2:** Number of clients ages 18–29 years who initiated PrEP at AHF and the proportion who received second, third, and fourth prescriptions by race/ethnicity, pre-COVID-19 versus post-COVID-19.

	Race/Ethnicity			
PrEP Prescription	White N (% of Initiation)	Non-White N (% of Initiation)	Unknown Race/Ethnicity N (% of Initiation)	Total N (% of Initiation)
Pre-COVID-19				
Initiation	310 (100.0)	475 (100.0)	646 (100.0)	1431 (100.0)
Second Prescription	305 (98.4)	467 (98.3)	586 (90.7)	1358 (94.9)
Third Prescription	299 (96.5)	451 (94.9)	561 (86.8)	1311 (91.6)
Fourth Prescription	296 (95.5)	441 (92.8)	496 (76.8)	1233 (86.2)
Post-COVID-19				
Initiation	1080 (100.0)	2686 (100.0)	850 (100.0)	4616 (100.0)
Second Prescription	965 (89.4)	2316 (86.2)	740 (87.1)	4021 (87.1)
Third Prescription	855 (79.2)	2005 (74.6)	631 (74.2)	3491 (75.6)
Fourth Prescription	717 (66.4)	1643 (61.2)	528 (62.1)	2888 (62.6)
Total				
Initiation	1390 (100.0)	3161 (100.0)	1496 (100.0)	6047 (100.0)
Second Prescription	1270 (91.4)	2783 (88.0)	1326 (88.6)	5379 (89.0)
Third Prescription	1154 (83.0)	2456 (77.7)	1192 (79.7)	4802 (79.4)
Fourth Prescription	1013 (72.9)	2084 (65.9)	1024 (68.4)	4121 (68.1)

**Table 3:** Logistic regression predicting odds of PrEP retention by the fourth prescription among clients ages 18–29 years who initiated PrEP at AHF by race/ethnicity.

	Odds Ratio (OR)	95% Confidence Interval (CI)	*p*-Value
Time Period			
Pre-COVID-19 (Reference)	—	—	—
Post-COVID-19	0.13	0.09–0.18	*p* < 0.001
Race/Ethnicity			
White (Reference)	—	—	—
Non-White	0.80	0.69–0.93	*p* = 0.003
Interaction Term			
Race/Ethnicity × Time Period	1.34	0.70–2.58	*p* = 0.379

## Data Availability

The data presented in this article are not publicly available due to client confidentiality and restrictions imposed by AIDS Healthcare Foundation (AHF). Requests to access the data will be reviewed on a case-by-case basis and may be considered in accordance with organizational policies and applicable regulations.

## Abbreviations

The following abbreviations are used in this manuscript:

AHF	AIDS Healthcare Foundation
CDC	Centers for Disease Control and Prevention
CI	Confidence Interval
COVID-19	Coronavirus Disease 2019
D.C.	District of Columbia
OR	Odds Ratio
PrEP	Pre-Exposure Prophylaxis
U.S.	United States

## References

[1] Centers for Disease Control and Prevention (CDC) (2025). Clinical Guidance for PrEP. https://www.cdc.gov/hivnexus/hcp/prep/index.html.

[2] Centers for Disease Control and Prevention (CDC), National Center for HIV, Viral Hepatitis, STD, and Tuberculosis Prevention (NCHHSTP) (2023). Expanding PrEP Coverage in the United States to Achieve EHE Goals. https://www.cdc.gov/nchhstp/director-letters/expanding-prep-coverage.html.

[3] Burns C.M., Borges M., Frye J., Keicher K., Elliott S., Schwartz S., Shipp K., Okeke N.L., McKellar M.S. (2022). Understanding retention in pre-exposure prophylaxis care in the South: Insights from an academic HIV prevention clinic. AIDS Res. Hum. Retroviruses.

[4] Chan P.A., Mena L., Patel R., Oldenburg C.E., Beauchamps L., Perez-Brumer A.G., Parker S., Mayer K.H., Mimiaga M.J., Nunn A. (2016). Retention in care outcomes for HIV pre-exposure prophylaxis implementation programmes among men who have sex with men in three US cities. J. Int. AIDS Soc..

[5] Agarwal H., Erwin M., Lyles S., Esposito M., Ahsan Z. (2024). Lower PrEP retention among young and Black clients accessing PrEP at a cluster of safety net clinics for gay and bisexual men. J. Int. Assoc. Provid. AIDS Care.

[6] Hernes S., Panwala R., Pfeil A., Sardinha M., Rossi V., Blumenthal J., Hill L. (2023). Predictors of PrEP retention in at risk patients seen at a HIV primary care clinic in San Diego. Int. J. STD AIDS.

[7] Krakower D.S., Maloney K.M., Powell V.E., Levine K., Grasso C., Melbourne K., Mayer K.H. (2022). Patterns and clinical consequences of discontinuing HIV preexposure prophylaxis during the COVID-19 pandemic. J. Acquir. Immune Defic. Syndr..

[8] Hong C. (2023). Characterizing the impact of the COVID-19 pandemic on HIV PrEP care: A review and synthesis of the literature. AIDS Behav..

[9] Huang Y.L.A., Zhu W., Wiener J., Kourtis A.P., Hall H.I., Hoover K.W. (2022). Impact of Coronavirus Disease 2019 (COVID-19) on human immunodeficiency virus (HIV) pre-exposure prophylaxis prescriptions in the United States—A time-series analysis. Clin. Infect. Dis..

[10] Chan P.A., Goedel W.C., Li Y., Mena L., Patel R.R., Marshall B.D.L., Yelena M., Ward L., Underwood A., Johnson C.J. (2025). Impact of Social Determinants of Health on Pre-Exposure Prophylaxis Care for HIV Prevention. J. Acquir. Immune Defic. Syndr..

[11] Hojilla J.C., Vlahov D., Crouch P.C., Dawson-Rose C., Freeborn K., Carrico A. (2018). HIV Pre-Exposure Prophylaxis (PrEP) Uptake and Retention among Men Who Have Sex with Men in a Community-Based Sexual Health Clinic. AIDS Behav..

[12] Rusie L.K., Orengo C., Burrell D., Ramachandran A., Houlberg M., Keglovitz K., Munar D., Schneider J.A. (2018). Preexposure Prophylaxis Initiation and Retention in Care over 5 Years, 2012–2017: Are Quarterly Visits Too Much?. Clin. Infect. Dis..

[13] Wu L., Schumacher C., Chandran A., Fields E., Price A., Greenbaum A., Jennings J.M., IMPACT Partner Collaborative (2020). Patterns of PrEP Retention among HIV Pre-Exposure Prophylaxis Users in Baltimore City, Maryland. J. Acquir. Immune Defic. Syndr..

[14] Patel V.V., Ogunbajo A., Willie T.C., Franks J., Sullivan P.S., Stephenson R. (2025). Changes in HIV pre-exposure prophylaxis use and care engagement among men who have sex with men during the COVID-19 pandemic. AIDS Behav..

[15] Daniels J., Kaur M., Ogunbajo A., Patel R.R., Khosropour C.M., Sullivan P.S., Siegler A.J. (2023). PrEP use and persistence in the United States before and after the COVID-19 pandemic: Evidence from a large national cohort. AIDS Behav..

[16] Smith D.K., Hosek S., Bekker L.G., Marrazzo J.M., Grant R.M. (2024). A systematic review and meta-analysis of HIV pre-exposure prophylaxis adherence and persistence across populations. J. Acquir. Immune Defic. Syndr..

[17] U.S. Food and Drug Administration (2019). FDA Approves Second Drug to Prevent HIV Infection as Part of Ongoing Efforts to End the HIV Epidemic. PR Newswire. https://www.prnewswire.com/news-releases/fda-approves-second-drug-to-prevent-hiv-infection-as-part-of-ongoing-efforts-to-end-the-hiv-epidemic-300930895.html.

[18] U.S. Food and Drug Administration (2021). FDA Approves First Injectable Treatment for HIV Pre-Exposure Prevention. FDA Press Announcements. https://www.fda.gov/news-events/press-announcements/fda-approves-first-injectable-treatment-hiv-pre-exposure-prevention.

[19] Centers for Disease Control and Prevention (CDC) Ending the HIV Epidemic in the U.S. Goals. https://www.cdc.gov/ehe/php/about/goals.html.

